# Differences in outcomes of combined heart-liver transplantation by primary cardiac diagnosis

**DOI:** 10.1016/j.jhlto.2024.100147

**Published:** 2024-08-12

**Authors:** Ye In Christopher Kwon, Emily Dunbar, Kelly Wright, Graham Gardner, Matthew Ambrosio, Inna F. Tchoukina, Keyur B. Shah, David Bruno, Amit Sharma, Josue Chery, Vigneshwar Kasirajan, Zubair A. Hashmi

**Affiliations:** a School of Medicine, Virginia Commonwealth University, Richmond, Virginia; b Division of General Surgery, Department of Surgery, Virginia Commonwealth University, Richmond, Virginia; c Department of Biostatistics, School of Population Health, Virginia Commonwealth University, Richmond, Virginia; d Division of Cardiology, Department of Internal Medicine, Virginia Commonwealth University, Richmond, Virginia; e Division of Abdominal Transplant Surgery, Department of Surgery, Hume-Lee Transplant Center, Virginia Commonwealth University, Richmond, Virginia; f Division of Cardiothoracic Surgery, Department of Surgery, Pauley Heart Center, Virginia Commonwealth University, Richmond, Virginia

**Keywords:** heart transplant, liver transplant, congenital heart disease, heart failure, Fontan procedure

## Abstract

**Background:**

Combined heart-liver transplantation (CHLT) is a complex procedure with rising demand and is subject to ongoing assessment. Here, we provide an update on indications, patient outcomes, and risk factors.

**Methods:**

This retrospective study utilized CHLT data from the United Network for Organ Sharing registry between 1990 and 2023. Recipient and donor characteristics, and risk factors for mortality were analyzed using Cox regression hazard models. Recipient and graft survival at 30 days, 1 year, and 5 years were analyzed using the Kaplan-Meier method.

**Results:**

This cohort included 532 patients with median survival of 16.9 years (SD: 1.09). The most common indications for CHLT were congenital heart disease (36%) and dilated cardiomyopathy (31%). Patient survival at 30 days, 1 year, and 5 years were 94%, 85%, and 77%, respectively. Combined heart-liver graft survival was 93%, 85%, and 77%, respectively. Diabetes (hazard ratio [HR]: 1.74; *p* = 0.04) was associated with multigraft failure and mortality in multivariate analysis. Compared to congenital heart disease, dilated (HR: 0.55; *p* = 0.03) and restrictive myopathies (HR: 0.5; *p* = 0.03) were associated with improved graft and overall survival. Higher donor left ventricular ejection fraction (EF) was also associated with improved graft and overall survival (HR: 0.96; *p* = 0.008).

**Conclusions:**

CHLTs are being performed at increasingly higher rates with comparable survival to single-organ transplants. Diabetes was associated with increased mortality. Recipient dilated or restrictive myopathies and higher donor EF were correlated with improved survival compared to congenital heart disease. Further studies are needed to better understand these observations.

## Background

Combined heart-liver transplantation (CHLT) is a sophisticated, life-saving procedure that treats often fatal diseases such as late-stage congenital heart disease (CHD) with subsequent irreversible liver dysfunction.^1^ In the United States, the demand for CHLTs has been rising with improved outcomes, particularly as a significant proportion of patients with congenital univentricular heart disease are now surviving into their adult years.^2^, ^3^ Many of these patients may have previously undergone a Fontan procedure.^4^, ^5^ Consequently, the damage to the end organs is believed to be caused by an interplay of elevated central venous pressure, low cardiac output, hepatic fibrosis, and hypoxia due to the progressive failure of the Fontan procedure.^3^, ^5^ In the absence of effective medical therapies for patients with simultaneous end-stage heart failure and decompensating Fontan-associated liver disease, CHLTs and isolated heart (IHT) or liver transplantation (ILT) remain the primary recourse.^6^ The higher likelihood of CHLT being a reoperation for adults with CHD presents unique challenges for these patients. IHT, particularly in the setting of concurrent end-stage liver disease, may not be a viable alternative given mortality rates as high as 50%.^7^ However, CHLT may offer much improved long-term survival benefits for congenital Fontan patients with up to 87%, 80%, and 78% 1-, 3-, and 5-year survival rates, respectively.^5^

While CHD remains the most common indication, there is increasing heterogeneity of pathologies that necessitate further evaluation for CHLT. Chronic liver disease can coexist with cardiomyopathy by many mechanisms, including alcohol use disorder, metabolic syndromes, or hepatitis C.^5^, ^8^ To our knowledge, the differences in patient outcomes between CHD and other cardiac indications for CHLT have not been clearly understood. Given the higher-risk profiles of adults with CHD, ongoing assessment of current perioperative practices and outcomes of CHLT is warranted to better guide patient evaluation, selection, and management. Here, we provide an updated retrospective cohort analysis on the differences in short- and long-term outcomes and risk factors for patients undergoing CHLT with different primary cardiac diagnoses in the United States between 1990 and 2023.

## Materials and methods

### Source of data

This study utilized the United Network for Organ Sharing (UNOS) Standard Analysis and Research database, which facilitates the Organ Procurement and Transplantation Network (OPTN). This is a prospectively maintained database of all candidates listed for solid-organ transplantation in the United States since 1987. This study followed the Strengthening The Reporting of Observational Studies in Epidemiology guidelines. As all data are deidentified, this retrospective study was deemed exempt from the Virginia Commonwealth University Institutional Review Board.

### Study population

The OPTN/UNOS Standard Analysis and Research file was queried to identify records of all adult patients (aged ≥18 years) listed for CHLT in the United States from January 1, 1990 to June 30, 2023. Patients who underwent multiorgan transplants were excluded. Patients were stratified into the following 4 primary cardiac diagnoses: CHD, dilated cardiomyopathy, restrictive cardiomyopathy, and others. We also stratified patients based on allocation eras. Era 1 was defined as January 1, 1990 to October 18, 2018. Era 2 was defined as October 19, 2018 to June 30, 2023.

### Statistical analysis

Comprehensive donor and recipient characteristics were collected, with categorical variables reported as percentages and continuous variables reported as medians and interquartile range (IQR). Pearson’s chi-square test or Fisher’s exact test was used for categorical variables while Kruskal-Wallis rank-sum test was performed for continuous variables. Patient survival at 30 days, 1 year, and 5 years was defined as the length of time from the date of CHLT until the date of last patient contact or death. Secondary outcomes were primary graft survival at 30-day, 1-, and 5-year intervals, length of hospital stay, rates of postoperative infection requiring hospitalization, and rates of acute rejection between transplant and discharge. The Kaplan-Meier method was used to plot and analyze overall survival for CHLT recipients, and 95% confidence intervals were reported for all outcomes. Between-indication and era comparisons were performed with the log-rank test. Multivariate Cox-proportional hazard regression was performed to assess significant predictors of overall survival. All statistical analyses were conducted using R (version 4.3.1). All *p*-values were based on 2-sided statistical tests and significance was set at *p* < 0.05.

## Results

### Recipient and donor differences by primary cardiac diagnosis

We identified 532 adult patients who underwent CHLT during the study period, among which 191 patients were diagnosed with CHD, 165 were diagnosed with dilated cardiomyopathy, and 98 were diagnosed with restrictive cardiomyopathy (Table 1). The average overall age of CHLT recipients was 47 years, whereas those with CHD tended to be younger at 33 years old at the time of transplant compared to those with dilated or restrictive cardiomyopathies (*p* < 0.001; Table 1). Overall time on the waitlist was 81.5 days and patients with CHD waited longer compared to those with dilated cardiomyopathy and other diagnoses (115 vs 49 vs 53.5 days; *p* < 0.001). However, patients with restrictive cardiomyopathy had the longest time on the waitlist at 121.5 days. Forty-six dilated cardiomyopathy patients had diabetes compared to 10 CHD patients and 9 restrictive cardiomyopathy patients (*p* < 0.001). CHD patients had the lowest body mass index (BMI) compared to dilated or ischemic cardiomyopathies and other diagnoses (23.7 vs 25.4 vs 25.3 vs 24.8; *p* = 0.002). About 96% of CHD patients underwent prior cardiac surgery whereas only 21% of patients with dilated cardiomyopathy and 11% of patients with ischemic cardiomyopathies underwent previous cardiac surgery (*p* < 0.001).

**Table 1:** Baseline Recipient and Donor Demographics and Characteristics by Diagnosis

Variables	Overall, N = 532^a^	Congenital heart disease, N = 191^a^	Dilated cardiomyopathy, N = 165^a^	Restrictive cardiomyopathy, N = 98^a^	Other, N = 78^a^	*p*-value^b^
*Recipient characteristics*						
Sex						<0.001
Female	162 (30%)	80 (42%)	45 (27%)	16 (16%)	21 (27%)	
Male	370 (70%)	111 (58%)	120 (73%)	82 (84%)	57 (73%)	
Age (IQR)	47.00 (34.00, 58.00)	33.00 (23.00, 42.00)	52.00 (44.00, 58.00)	59.00 (54.00, 63.00)	49.50 (40.25, 59.75)	<0.001
Waitlist days (IQR)	81.50 (31.00, 224.25)	115.00 (46.00, 283.50)	49.00 (21.00, 145.00)	121.50 (52.25, 272.00)	53.50 (24.25, 159.00)	<0.001
MELD-XI score at transplant (IQR)	15.00 (10.00, 19.00)	16.00 (11.00, 20.00)	15.00 (10.00, 20.50)	15.00 (11.00, 19.00)	14.00 (10.00, 18.00)	0.2
Serum creatinine at transplant (IQR)	1.10 (0.87, 1.41)	1.00 (0.78, 1.25)	1.20 (0.90, 1.49)	1.20 (0.91, 1.60)	1.10 (0.88, 1.40)	<0.001
eGFR at transplant (ml/min/1.73 m^2^) (IQR)	76.66 (55.52, 103.92)	90.07 (65.16, 118.72)	67.98 (49.74, 95.24)	69.04 (47.74, 90.72)	73.08 (58.43, 104.92)	<0.001
CKD stage at transplant (%)						0.2
Stage 1	192 (37%)	95 (50%)	46 (29%)	25 (26%)	26 (35%)	
Stage 2	170 (33%)	54 (29%)	56 (35%)	31 (32%)	29 (39%)	
Stage 3	137 (26%)	38 (20%)	48 (30%)	34 (35%)	17 (23%)	
Stage 4	18 (3.5%)	2 (1.1%)	7 (4.4%)	6 (6.3%)	3 (4.0%)	
Stage 5	1 (0.2%)	0 (0%)	1 (0.6%)	0 (0%)	0 (0%)	
Recipient diabetes at listing (%)	75 (14%)	10 (5.2%)	46 (28%)	9 (9.2%)	10 (13%)	<0.001
Cigarette use at listing (%)	147 (28%)	26 (14%)	68 (41%)	29 (30%)	24 (31%)	<0.001
Dialysis between listing and transplant (%)	21 (3.9%)	6 (3.1%)	9 (5.5%)	3 (3.1%)	3 (3.8%)	0.7
BMI at transplant (kg/m^2^) (IQR)	24.70 (21.90, 28.43)	23.70 (21.00, 27.10)	25.40 (23.10, 29.50)	25.30 (22.53, 28.38)	24.80 (22.08, 28.00)	0.002
On ventilator at transplant (%)	6 (1.1%)	3 (1.6%)	2 (1.2%)	1 (1.0%)	0 (0%)	>0.9
Serum bilirubin at transplant (mg/dl) (IQR)	1.00 (0.60, 1.60)	1.05 (0.60, 1.80)	1.00 (0.60, 1.50)	0.90 (0.68, 1.50)	1.00 (0.70, 1.90)	0.5
Serum albumin at transplant (g/dl)	3.80 (3.30, 4.30)	3.70 (3.10, 4.30)	3.60 (3.00, 3.90)	4.00 (3.70, 4.30)	3.85 (3.38, 4.25)	0.13
Prior cardiac surgery (%)	218 (49%)	157 (96%)	29 (21%)	9 (11%)	23 (34%)	<0.001
*Donor characteristics*						
Age (IQR)	27.00 (21.00, 36.00)	26.00 (20.00, 34.50)	29.00 (22.00, 38.00)	28.00 (21.25, 36.75)	26.00 (20.00, 38.00)	0.018
BMI (kg/m^2^) (IQR)	24.92 (22.42, 27.97)	24.45 (21.73, 27.54)	24.92 (22.65, 28.38)	25.83 (23.57, 27.97)	24.50 (21.85, 28.01)	0.078
Left ventricular ejection fraction (%) (IQR)	60.00 (58.00, 65.00)	60.00 (57.00, 65.00)	60.00 (57.75, 65.00)	60.00 (60.00, 65.00)	64.50 (59.00, 66.50)	0.5
Diabetes (%)	12 (2.3%)	3 (1.6%)	3 (1.8%)	2 (2.0%)	4 (5.1%)	0.4
Total cold ischemia time (hours) (IQR)	3.20 (2.50, 4.00)	3.75 (3.00, 4.80)	3.00 (2.30, 3.70)	2.80 (2.15, 3.40)	3.20 (2.40, 3.90)	<0.001

Abbreviations: BMI, body mass index; CKD, chronic kidney disease; eGFR, estimated glomerular filtration ratio; IQR, interquartile range; MELD-XI, model for end-stage liver disease excluding international normalized ratio.

a *n* (%); median (IQR).

b Pearson’s chi-squared tests; Kruskal-Wallis rank-sum test; Fisher’s exact test

Several differences in donor characteristics were identified between recipient diagnoses (Table 1). CHD patients received organs from the youngest donor at 26 years old compared to dilated (29 years) and restrictive (28) cardiomyopathy patients (*p* = 0.018). Overall cold ischemia time for the primary cardiac allograft was 3.2 hours. However, CHD patients experienced the longest cold ischemia time at 3.75 hours compared to those with dilated (3 hours) and restrictive (2.8 hours) cardiomyopathies (*p* < 0.001). We observed no differences in donor left ventricular ejection fraction (LVEF), BMI, or concurrent diabetic status across recipient cardiac diagnoses groups.

### Recipient and donor differences by transplant era

**Figure 1:**
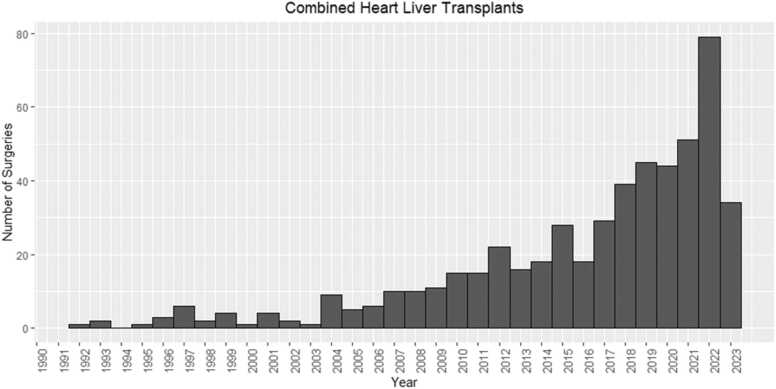
Trends in the number of combined heart-liver transplants performed in the United States by year.

The number of CHLTs performed in the United States has been rising during the study period (Figure 1). Between 1990 and 2018, 268 CHLTs were performed, whereas 264 cases have already been performed within 6 years since 2018 (Table 2). However, in era 2, 49% of recipients were diagnosed with CHD compared to 23% in era 1 (*p* < 0.001). Recipients in era 2 were younger (*p* < 0.001), had shorter waitlist days (*p* < 0.001), less likely to have smoked cigarettes (*p* < 0.001), and had undergone more prior cardiac surgery (*p* < 0.001). Most recipients in era 1 were listed at status 1A (38%) or 1B (36%), whereas most recipients in era 2 were listed as status 2 (40%) followed by status 4 (37%). Donors in era 2 had significantly lower LVEF (*p* = 0.013), yet higher ischemic times (*p* < 0.001) compared to those in era 1. Importantly, recipients in era 2 were more likely to be supported on intra-aortic balloon pumps (IABP) compared to those in era 1 (*p* = 0.012).

**Table 2:** Baseline Recipient and Donor Demographics and Characteristics by Transplant Eras

Variables	Overall, N = 532^a^	Era 1, N = 268^a^	Era 2, N = 264^a^	*p*-value^b^
*Recipient characteristics*				
Diagnosis (%)				<0.001
Congenital heart disease	191 (36)	62 (23)	129 (49)	
Dilated cardiomyopathy	165 (31)	82 (31)	83 (31)	
Restrictive cardiomyopathy	98 (18)	79 (29)	19 (7.2)	
Other	78 (15)	45 (17)	33 (13)	
Sex (%)				0.4
Female	162 (30)	77 (29)	85 (32)	
Male	370 (70)	191 (71)	179 (68)	
Age (IQR)	47.00 (34.00, 58.00)	49.00 (37.00, 59.00)	44.50 (31.75, 55.00)	<0.001
Waitlist days (IQR)	81.50 (31.00, 224.25)	106.50 (46.00, 271.00)	61.50 (23.50, 193.00)	<0.001
MELD-XI score at transplant (IQR)	15.00 (10.00, 19.00)	15.00 (10.00, 19.00)	15.00 (10.00, 21.00)	0.12
Serum creatinine at transplant (IQR)	1.10 (0.87, 1.41)	1.10 (0.90, 1.50)	1.10 (0.83, 1.38)	0.11
eGFR at transplant (ml/min/1.73 m^2^) (IQR)	76.66 (55.52, 103.92)	73.99 (53.28, 101.67)	78.72 (59.01, 108.38)	0.031
CKD stage at transplant (%)				0.2
Stage 1	192 (37)	89 (34)	103 (41)	
Stage 2	170 (33)	86 (33)	84 (33)	
Stage 3	137 (26)	78 (30)	59 (23)	
Stage 4	18 (3.5)	11 (4.2)	7 (2.8)	
Stage 5	1 (0.2)	0 (0)	1 (0.4)	
Diabetes at listing (%)	75 (14)	33 (12)	42 (16)	0.2
Cigarette use at listing (%)	147 (28)	87 (32)	60 (23)	<0.001
Dialysis between listing and transplant (%)	21 (3.9)	14 (5.2)	7 (2.7)	0.13
BMI (IQR)	24.70 (21.90, 28.43)	24.40 (21.88, 28.03)	24.80 (21.90, 28.80)	0.3
On ventilator at transplant (%)	6 (1.1%)	3 (1.1%)	3 (1.1%)	>0.9
Ventricular assist devices at transplant (%)	23 (4.3)	11 (4.2)	12 (4.5)	0.57
Intra-aortic balloon pump at transplant (%)		11 (4.1)	26 (9.8)	0.012
Serum bilirubin at transplant (mg/dl) (IQR)	1.00 (0.60, 1.60)	1.00 (0.60, 1.60)	1.00 (0.60, 1.70)	0.7
Serum albumin at transplant (g/dl) (IQR)	3.80 (3.30, 4.30)	3.80 (3.28, 4.30)	3.90 (3.50, 4.35)	0.3
Prior cardiac surgery (%)	218 (49)	80 (36)	138 (61)	<0.001
Allocation status in era 1 (%)				
1A	N/A	99 (38)	N/A	
1B	N/A	92 (36)	N/A	
2	N/A	67 (26)	N/A	
Allocation status in era 2 (%)				
1	N/A	N/A	11 (4.2)	
2	N/A	N/A	105 (40)	
3	N/A	N/A	37 (14)	
4	N/A	N/A	97 (37)	
5	N/A	N/A	13 (4.9)	
6			1 (0.4)	
*Donor characteristics*				
Age (IQR)	27.00 (21.00, 36.00)	26.50 (20.00, 37.00)	28.00 (22.00, 36.00)	0.5
BMI (kg/m^2^) (IQR)	24.92 (22.42, 27.97)	24.92 (22.67, 27.45)	24.85 (22.25, 28.68)	0.5
Left ventricular ejection fraction (%) (IQR)	60.00 (58.00, 65.00)	64.00 (60.00, 65.00)	60.00 (56.00, 65.00)	0.013
Diabetes (%)	12 (2.3)	8 (3.0)	4 (1.5)	0.3
Total cold ischemia time (hours) (IQR)	3.20 (2.50, 4.00)	2.90 (2.13, 3.68)	3.60 (3.00, 4.40)	<0.001

Abbreviations: BMI, body mass index; CKD, chronic kidney disease; eGFR, estimated glomerular filtration ratio; IQR, interquartile range; MELD-XI, model for end-stage liver disease excluding international normalized ratio.

a *n* (%); median (IQR).

b Pearson’s chi-squared test; Wilcoxon rank-sum test; Fisher’s exact test.

### Clinical outcomes

**Figure 2:**
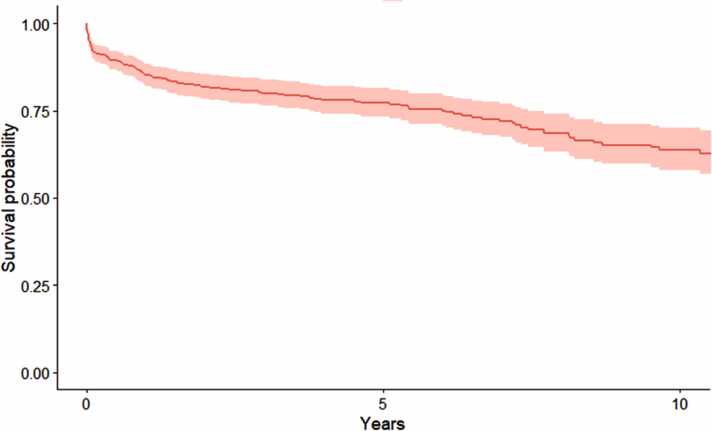
Kaplan-Meier survival curve for all recipients of combined heart-liver transplantation. Shaded region represents a 95% confidence interval.

The 30-day, 1-, and 5-year overall survival for all adult patients undergoing CHLT were 93.8%, 85.1%, and 77.3%, respectively. Combined cardiac and hepatic graft survival for all adult patients undergoing CHLT were 93.2%, 84.6%, and 77% at 30 days, 1, and 5 years, respectively. Overall rate of postoperative infection requiring hospitalization was 23.7% whereas only 5.26% of the whole cohort experienced acute rejection. The median length of stay for the whole cohort was 24 days (Table 3). Kaplan-Meier survival curve also revealed that 10-year survival for the whole cohort is projected to be over 65% (Figure 2).

**Table 3:** Overall Outcomes of All Adults Undergoing Combined Heart-Liver Transplantation

Outcomes of interest	Percent (95% CI)
Postoperative infection requiring hospitalization (%)	23.7
Acute rejection (%)	5.26
Length of hospital stay (days) (95% CI)	24 (16-38)
30-day survival (%) (95% CI)	93.8 (91.7-95.9)
1-year survival (%) (95% CI)	85.1 (82.0-88.3)
5-year survival (%) (95% CI)	77.3 (73.3-81.4)
30-day graft survival (%) (95% CI)	93.2 (91.0-95.4)
1-year graft survival (%) (95% CI)	84.6 (81.5-87.9)
5-year graft survival (%) (95% CI)	77.0 (73.1-81.2)

Abbreviation: CI, confidence interval.

**Figure 3:**
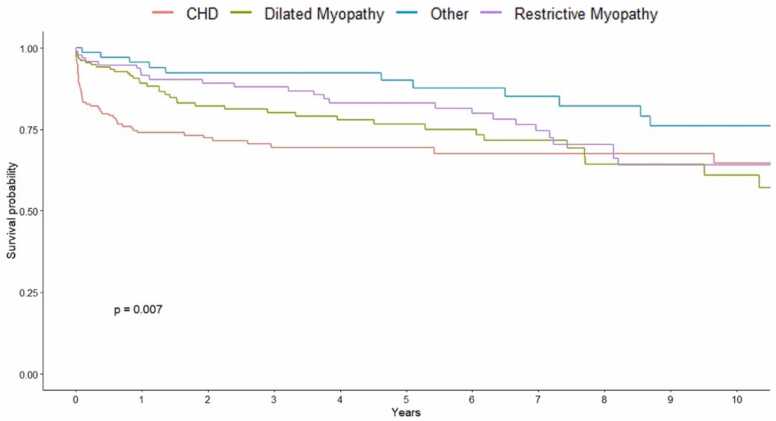
Kaplan-Meier survival curve demonstrating differences in short-, medium-, and long-term survival by cardiac diagnosis and indication for combined heart-liver transplantation. CHD, congenital heart disease.

**Figure 4:**
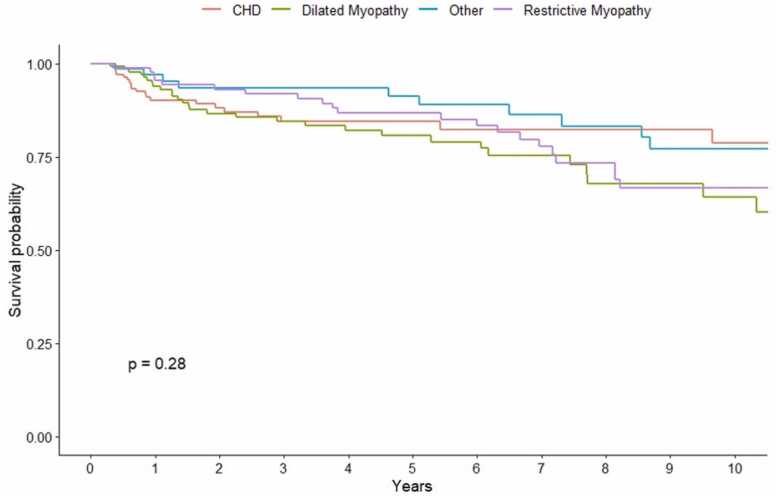
Conditional Kaplan-Meier survival curve demonstrating differences in survival by primary cardiac diagnoses excluding patients who died or lost to follow-up in the first 90 days post-CHLT. CHD, congenital heart disease; CHLT, combined heart-liver transplantation.

**Figure 5:**
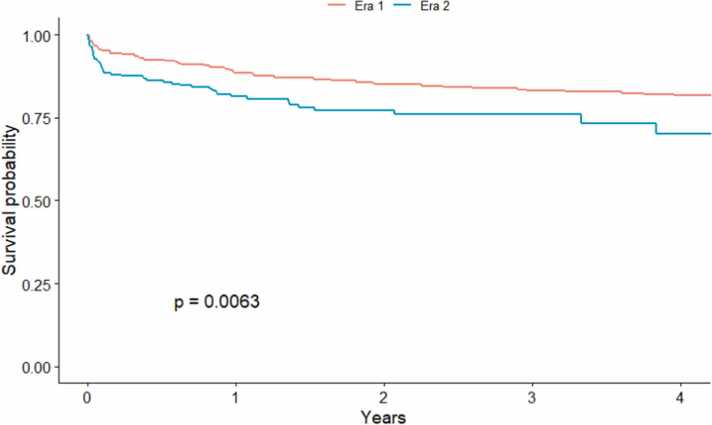
Kaplan-Meier survival curve comparing the overall survival for all combined heart-liver transplantation between 2 eras.

**Figure 6:**
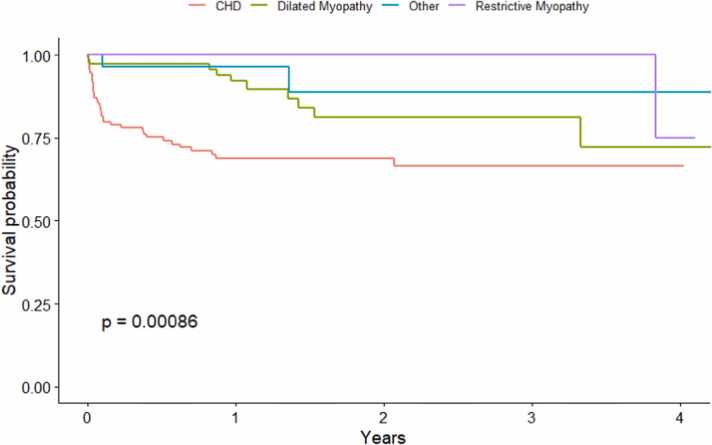
Kaplan-Meier survival curve comparing the overall survival between cardiac diagnoses within era 2. CHD, congenital heart disease.

When analyzed by allocation eras, significant differences in short- and long-term survival rates were observed between primary cardiac diagnoses (*p* = 0.017; Figure 3). Conditional survival analysis excluding patients who died or lost to follow-up before the first 90 days post-CHLT revealed comparable survival between primary cardiac diagnoses at 1-, 5-, and 10-year intervals (*p* = 0.28; Figure 4; Table S1). There were significant differences in survival between allocation eras (*p* = 0.0063; Figure 5). While we observed no differences in short- and long-term survival between recipients of varying cardiac diagnoses within era 1 (*p* = 0.13; Figure S1), patients with CHD had significantly lower survival rates compared to those with other cardiomyopathies in era 2 (*p* = 0.00086; Figure 6).

Multivariate Cox regression analysis (Table 4) revealed that recipient diabetic status at listing was associated with increased rates of overall mortality (hazard ratio [HR] = 1.74; *p* = 0.04). Compared to CHD as an indication for CHLT, diagnoses as dilated (HR = 0.55; *p* = 0.03) and restrictive (HR = 0.5; *p* = 0.03) cardiomyopathy were associated with improved multigraft and overall survival. Conversely, higher donor LVEF was associated with improved multigraft and overall patient survival (HR = 0.96; *p* = 0.008). CHLT in allocation era 2 was associated with increased risk of mortality (HR = 1.58; *p* = 0.048) compared to those in era 1. In era 1, allocation status was not an independent predictor of mortality. However, in era 2, status 2 (HR = 0.16; *p* = 0.008), 3 (HR = 0.15; *p* = 0.013), and 4 (HR = 0.18; *p* = 0.013) patients had significantly lower risk of mortality.

**Table 4:** Cox-Proportional Hazard Model for Overall Survival for All Adults Undergoing Combined Heart-Liver Transplantation

Predictors	Hazard ratio	*p-*value
Sex (male)	1.08	0.72
Age	1.00	0.60
Diabetes	1.74	0.04
Cigarette use	1.06	0.78
MELD-XI	0.99	0.37
Diagnosis (congenital heart disease as reference)		
Dilated cardiomyopathy	0.55	0.03
Restrictive cardiomyopathy	0.50	0.03
Other	0.25	<0.001
Donor age	1.00	0.87
Ejection fraction	0.96	0.008
Era 2 (era 1 as reference)	1.58	0.048
Allocation status in era 1 (status 1A as reference)		
Status 1B	1.19	0.539
Status 2	0.79	0.527
Allocation status in era 2 (status 1 as reference)		
Status 2	0.16	0.008
Status 3	0.15	0.013
Status 4	0.18	0.013
Status 5	0.36	0.231
Status 6	0.00	0.997

Abbreviation: MELD-XI, model for end-stage liver disease excluding international normalized ratio.

## Discussion

Between 1990 and 2023, CHLTs among adult patients continue to be performed at increasingly higher rates annually, with observed sharp increases since 2018. The year 2022 recorded the highest number of CHLTs performed, likely due to the backlog of elective surgeries accrued during the COVID-19 pandemic.^9^ Notably, there has been an evolution of indications for this procedure. Since the implementation of the 6-tiered donor heart allocation system in 2018 designed to prioritize patients on mechanical circulatory support (MCS),^10^ CHD has become the predominant indication for CHLT. This is despite the relatively limited durable MCS options and challenging anatomic considerations.^11^ The increased utilization of IABP support, which provides status 2 under the new allocation system,^12^ demonstrates the potential advantages of IABP over ventricular assist device (VAD) among CHLT recipients. The compact design, absence of venous cannulation, and different strategies for access render IABP a relatively more appealing option.^13^, ^14^ This shift also aligns with the increasing prevalence of liver disease in younger patients with univentricular physiology who have previously undergone palliative Fontan procedures.^3^ A recent analysis showed that, especially after small conduit extracardiac Fontan, prevalence of cirrhosis within 15 years neared 30%.^15^ Our data support this notion with most CHD patients having undergone prior cardiac surgery, particularly since 2018. As the life expectancy following Fontan procedure extends beyond 25 years, this presents an extended window of opportunity for many of these CHD patients to develop severe heart failure secondary to Fontan-associated liver disease.^3^, ^16^ Even in hemodynamically stable patients, performing ILT has been discouraged due to the challenges in managing elevated right-sided pressures during the anhepatic and reperfusion phases.^17^

The lower survival rates for CHLT recipients in the modern era appear to be largely driven by the increased mortality among CHD recipients in the modern era. While CHD patients may be younger, less likely to be diabetic and less likely to have smoked cigarettes, they also experienced longer days on waitlist. Historically, CHD patients have had increased mortality on the waitlist and were less likely to be bridged with MCS than patients with other etiologies for heart failure.^18^ In addition, the expanded regional sharing for patients with new status 1 and 2 may have a further impact on allocation for dual-organ recipients.^12^ There may be some preferential distribution of organs to patients with single-organ disease on temporary MCS, rather than CHD patients with multiorgan diseases and limited MCS options.^13^ However, this discrepancy may also be attributed to the similarly lower mid- and long-term survival seen in the “other indications” group.

Contrary to previous analyses, our updated analysis found cardiac diagnoses to be associated with overall survival.^5^ While our study focused on the interaction between primary cardiac diagnosis and post-CHLT outcomes, it should be noted that up to 50% of patients with end-stage liver disease may develop cirrhotic cardiomyopathy.^8^ Whether end-stage liver failure plays a role in CHLT outcomes warrants future exploration. Nevertheless, the primary challenge in evaluating candidates for CHLT appears to be determining how sick the liver is in the setting of end-stage heart failure in CHD patients. The long-term survival after Fontan procedure in CHD patients is suboptimal with 74%, 61%, and 43% at 10, 20, and 30 years, respectively.^19^ While these patients may be surviving into adulthood, their baseline survival may have already been lowered due to the surgical complications introduced during their initial Fontan procedure. Even among higher-risk CHD patients who have undergone a Fontan procedure, our data demonstrate comparable mid- to long-term survival profiles for all CHLT recipients to IHT or ILT.^2^, ^20^

Furthermore, much of the morbidity may be attributed to the upfront surgical risk. When excluding the first 90 days after CHLT, 1-year survival did not differ significantly between cardiac diagnoses. The observed worse early outcomes for CHD patients may be attributed to longer cold ischemic times experienced by their respective donor organs. Recent studies found that donor heart ischemic time threshold for survival occurs at 3 to 4 hours, suggesting the need to use different myocardial preservation strategies if the expected cold ischemia time surpasses that mark.^21^, ^22^, ^23^ While the increased median ischemic time of 3.75 hours for donor organs allotted to CHD recipients may be under the proposed 4-hour cut-off, it may have consequences affecting survival outcomes. Nevertheless, with the emergence of new technologies such as the SherpaPak Cardiac Transportation System or XVIVO Heart/Liver Assist Transport, cold ischemic times for CHLT may also be considerably extended beyond the 4-hour limit with comparable outcomes.^24^, ^25^, ^26^ In addition, the minimal use of VADs in the post-2018 era may have allowed CHD patients to benefit from the reduced prioritization of patients supported on durable VADs.^13^ Akin to prior studies, prolonged ischemic times appear to align with the increased geographical sharing available to patients newly listed as status 1 or 2, resulting in comparatively shorter waitlist times.^13^ In general, our results suggest that higher allocation status may have long-term benefits on survival for CHD patients.

Additionally, recipient’s diabetic status continued to be associated with increased mortality and risk for earlier onset multigraft failure, further indicating the significant burden that preoperative diabetes-associated organ damage represents on postoperative outcomes among CHLT recipients. On the other hand, higher donor LVEF was associated with improved outcomes. However, other studies have suggested that when evaluating 1-year survivors, donor LVEF was not associated with superior 10-year outcomes.^27^ This is not unexpected given that a singular metric, such as ejection fraction, might not provide a precise indication of the quality of the allograft.^27^

The role of transplant techniques on short-term outcomes merits further investigation in future studies. The most described technique is performing the heart transplant on cardiopulmonary bypass (CPB) followed by performing the liver transplant on venovenous bypass. This is particularly known to be useful for patients with familial amyloid polyneuropathy. However, venovenous bypass may be completely avoided through the “piggyback” technique during which the mediastinum is left open throughout liver transplant.^28^, ^29^ Some centers have used the en-bloc technique allowing both organs to be perfused simultaneously.^29^, ^30^ This may reduce the hepatic cold ischemic time and minimize hemodynamic impact of reperfusion since CPB supports both organs simultaneously.^30^ However, simultaneous reperfusion may increase cross-clamp time on CPB. Therefore, in most cases, it is not a standard practice.^31^ In our experience, continuing CPB with partial flow during the liver transplant appears to provide the much-needed hemodynamic support to the cardiac allograft while allowing some protection against reperfusion injury to the liver. These wide technical variations present challenges to evaluate the most optimal operative strategies for CHLT. Therefore, this study further underscores the significance of meticulous patient and donor selection for CHLT considering the unique attributes of each patient and donor, alongside the necessity for strategic preoperative preparation and risk stratification among surgeons specializing in both heart and liver procedures.

## Limitations

While we present the most updated, nationwide analysis of CHLTs performed over the past 3 decades, certain limitations should be considered when interpreting our findings. First, the retrospective nature of our study introduces a level of selection bias. However, we attempted to mitigate this bias by including all adult patients listed for CHLT regardless of procurement methodology, waitlist status, or transplant center volume. Second, there may be inconsistencies and lack of granularity in data that could impact patient survival, particularly in the number of previous surgeries undergone in CHD patients and the instances where a liver transplant was aborted due to unknown intraoperative issues induced by the heart transplant. Third, the lack of statistical significance in certain variables may be due to the insufficient power to identify a potential association.

Finally, the UNOS database does not have direct qualifiers to determine the sequence of CHLT. Thus, it was not possible for our study to accurately discern the specific reasons why patients underwent heart or liver transplant first or both organs simultaneously. Previous studies have attempted to determine transplant sequences by subtracting the cardiac cold ischemia time from the liver cold ischemia time.^5^ However, this does not consider the possibility of either organs originating from different donors as not all cases are likely to have been from a single donor or used a more selective, en-bloc technique for procurement and transplantation. Thus, we opted to exclude this analysis. UNOS/OPTN should collect more detailed data regarding the sequence of transplant in these rarer double organ procedures to aid our evaluation of the limits of multiorgan ischemia. Additional metrics such as anatomical variations, biliary complications, and the extent of liver cirrhosis by computed tomography findings will be needed to better understand the underlying mechanism behind differences in outcomes of CHLT between primary cardiac diagnoses.

## Conclusion

This study provides the most recent analysis on the trends and outcomes of CHLTs among adult recipients in the United States between 1990 and 2023. CHLTs continue to be performed at increasingly higher rates with comparable survival to IHT or ILT. Significant differences in clinical characteristics and outcomes persist between recipients with different primary cardiac indications for CHLT. Patients with CHD, while making up the majority of CHLT recipients in the post-2018 allocation system era, continue to represent higher risks associated with worse outcomes after CHLT. However, after the first 90 days, CHD patients have comparably favorable survival compared to other indications. Taken together, we present an update on multidisciplinary factors that may better guide appropriate recipient and donor selection and clinical outcomes for CHLT.

## Author contributions

Y.C.K., E.D., and Z.A.H. conceived and designed the study. Y.C.K. and E.D. contributed equally to the production and editing of this manuscript. M.A. contributed to the data and statistical analyses. K.W., R.D., G.G., I.F.T., K.B.S., A.S., D.B., J.C., and Z.A.H. reviewed and edited this manuscript. All authors agreed and approved of the contents of this manuscript.

## Disclosure statement

The authors declare that they have no known competing financial interests or personal relationships that could have appeared to influence the work reported in this paper.

We specially thank Matthew Ambrosio in the Department of Biostatistics for excellent statistical and data analysis support. We would like to thank the Pauley Heart Center for research support.

There were no outside sources of funding for this study.
