# Supplementary material for: Differences in outcomes of combined heart-liver transplantation by primary cardiac diagnosis
Source: JHLT Open. 2024 Aug 12;6:100147. doi: 10.1016/j.jhlto.2024.100147 (PMC11935517; doi:10.1016/j.jhlto.2024.100147)
Supplement: Supplementary file 1 — Supplementary material [file mmc1.docx]

**Supplementary Tables:**

Table S1. Conditional survival outcomes of adult patients undergoing combined heart-liver transplantation excluding those who died or lost to follow-up in the first 90 days.

| **Outcomes of Interest** | **Congenital Heart Disease, N = 143** | **Dilated Cardiomyopathy, N = 143** | **Restrictive Cardiomyopathy, N = 92** | **Other, N = 69** |
| --- | --- | --- | --- | --- |
| 1 Year Survival (%)  (95% CI) | 90.1 (85.2 - 95.4) | 94.0 (90.0 - 98.1) | 95.5 (91.3% - 99.9) | 97.0 (93.0 - 100) |
| 5 Year Survival (%)  (95% CI) | 84.6 (78.1 - 91.2) | 80.1 (73.4 - 88.7) | 86.7 (79.8 - 94.4) | 91.4 (84.3 - 99.0) |
| 10 Year Survival (%)  (95% CI) | 78.7 (69.0 - 89.6) | 64.2 (52.4 - 78.6) | 66.8 (55.5 - 80.4) | 77.1 (65.1 - 91.4) |


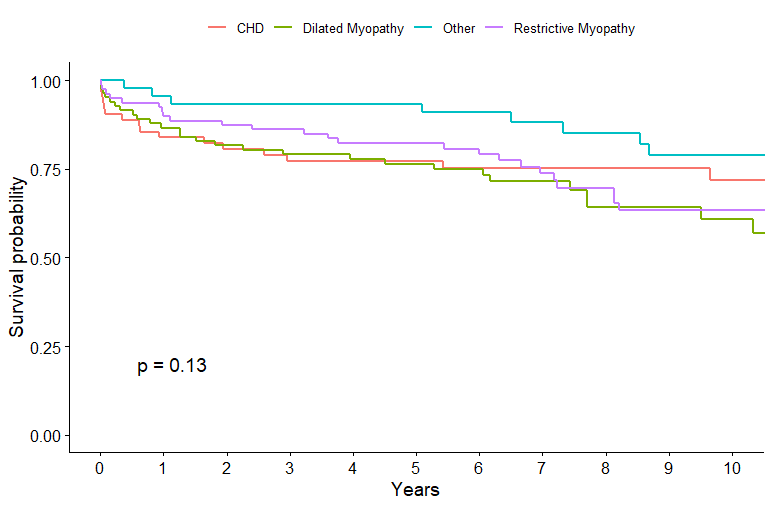


Figure S1. Kaplan-Meier survival curve comparing the overall survival between cardiac diagnoses within era 1.
